# Accuracy of the visual assessment of patellar tracking is poor and is influenced by trochlear dysplasia

**DOI:** 10.1002/jeo2.70487

**Published:** 2025-10-31

**Authors:** Alexander Bumberger, Andrew J. Cosgarea, Petri J. Sillanpaa, Matthew J. Best, Miho J. Tanaka

**Affiliations:** ^1^ Department of Orthopedics and Trauma Surgery Medical University of Vienna Vienna Austria; ^2^ Department of Orthopedic Surgery, Brigham and Women's Hospital Harvard Medical School Boston Massachusetts USA; ^3^ Department of Orthopaedic Surgery The Johns Hopkins Hospital Baltimore Maryland USA; ^4^ Department of Orthopaedic Surgery Pihlajalinna Hospital Tampere Finland; ^5^ Department of Orthopedic Surgery, Massachusetts General Hospital Harvard Medical School Boston Massachusetts USA

**Keywords:** 4D CT, dynamic imaging, J‐sign, patellofemoral instability, patellar tracking

## Abstract

**Purpose:**

The J‐sign is commonly used to assess patellar tracking in patellofemoral instability, though reliability is limited. This study analysed the diagnostic performance and reliability of digitally aided visual J‐sign assessments by patellofemoral specialists compared to dynamic kinematic computed tomography scans (DKCT).

**Methods:**

A diagnostic study was conducted with 20 experts assessing maltracking (≥2 quadrants lateral translation) in 17 standardised single‐knee videos of patients with and without patellofemoral instability. A digital reference line was added to guide visualisation. Experts performed qualitative (binary) assessments and quantitative gradings (0–3) of patellar tracking. Inter‐ and intra‐rater reliability was evaluated using kappa statistics. Diagnostic performance was compared to DKCT‐based patellar lateralisation (bisect offset [BO]). Logistic regression analysed the influence of tibial tuberosity–trochlear groove (TTTG) distance, lateral trochlear inclination (LTI) and Caton–Deschamps Index (CDI) on accuracy.

**Results:**

Moderate inter‐rater (*κ *= 0.52) and substantial intra‐rater (*κ* = 0.71) reliability were found for binary assessments, while quantitative grading showed substantial inter‐rater (*κ *= 0.62) and intra‐rater (*κ *= 0.69) reliability. Mean accuracy for binary assessments was 70.6%, whereas grading accuracy was lower at 36.8%, with variability across grades. LTI significantly influenced accuracy (*β* = 0.19, *p *= 0.048), with a 20.9% increase in correct diagnoses per degree of LTI. TTTG and CDI had no significant effect.

**Conclusion:**

Despite substantial intra‐rater and moderate inter‐rater reliability, accuracy in grading patellar tracking was low, particularly in quantitative assessments. Trochlear dysplasia affected diagnostic accuracy, suggesting the J‐sign is less reliable in dysplastic patients. More advanced imaging methods may be necessary to improve patellar tracking evaluations.

**Level of Evidence:**

Level III, diagnostic study.

**Abbreviations**
BO: bisect offset
CDI: Caton–Deschamps Index
DKCT: dynamic kinematic computed tomography
IPSG: International Patellofemoral Study Group
LTI: lateral trochlear inclination
MPFL: medial patello‐femoral ligament
TTPCL: tibial tubercle posterior cruciate ligament distance
TTTG: tibial tubercle–trochlear groove distance

## INTRODUCTION

The evaluation of patients with patellar instability can be complex due to the range of parameters that need to be considered. Relevant anatomic abnormalities, such as high‐grade trochlear dysplasia, patella alta, or an increased tibial tuberosity–trochlear groove (TTTG) distance, frequently lead to patellofemoral maltracking. Patellofemoral maltracking is defined as excessive lateral translation of the patella by more than two quadrants and is clinically referred to as ‘J‐sign’ when it occurs during terminal knee extension [6, 12, 14, 26].

The presence of a J‐sign on physical examination is regarded as an important finding that can guide surgical planning. Specifically, a high‐grade J‐sign has been identified as a significant predictor of inferior clinical outcomes following medial patellofemoral ligament reconstruction and therefore may indicate the need for additional bony procedures [5, 6, 9, 10, 14, 15, 22, 23, 27, 28]. The grading of a potential J‐sign as an indicator of patellar maltracking has traditionally been based on a visual assessment of the degree of subluxation of the patella in full active knee extension. Advanced imaging techniques such as dynamic kinematic computed tomography scans (DKCT) have allowed for a standardised quantification and grading of patellar maltracking by assessing the bisect offset (BO), a measure of patellar lateralisation, during active knee range of motion [2, 13, 17, 19, 24].

Previous literature has reported varying accuracy and inter‐reliability regarding visual grading of the J‐sign [1, 2, 8, 21]. A recent study demonstrated that certain morphological factors are associated with a higher‐grade J‐sign on visual assessment, but an objective reference standard was not available [21]. Overall, there is a paucity of data regarding the influence of morphological factors on the accuracy of visual assessments as compared to objective measurements. A more detailed understanding of when the J‐sign can be reliably and accurately assessed is crucial, given its high clinical relevance but poor reliability and accuracy on average.

The primary aim of this study was to determine the diagnostic performance and reliability of visual assessments of patellar tracking by surgeons using a novel digital reference line on knee videos to enhance the precision of visual assessments when compared with objective DKCT‐based measures. The secondary aim was to examine whether there was an association between morphological parameters of patellofemoral tracking and the accuracy of visual assessments. The hypothesis was that visual assessments would show varying diagnostic performance depending on established parameters of patellofemoral tracking.

## METHODS

This study received approval from our institutional review board (ID 2019P003614).

### Study design

This was a diagnostic study involving experts on patellofemoral instability who assessed single knee videos of patients with patellofemoral instability. Fourteen consecutive patients (17 knees) aged 18–34 years, who were evaluated for unilateral or bilateral patellar instability by two study authors (M.J.T. and A.J.C.) between 2015 and 2016, were enrolled. All patients had undergone DKCT of both knees as part of their diagnostic evaluation for recurrent patellar instability. Patients with incomplete DKCT imaging or those with poor image quality or lack of full extension were excluded from the study. Furthermore, patients with prior surgeries, severe osteoarthritis (Kellgren–Lawrence >2), and limited range of motion that did not allow for assessment of maltracking were excluded.

### Dynamic imaging

DKCT imaging was performed with a kinematic CT scanner (Toshiba Aquilion ONE; Canon Medical Systems USA, Inc), validated for assessing patellar tracking and measuring BO [18, 19, 25]. While seated in the CT gantry, participants performed active knee extension and flexion for two cycles, with CT images acquired at 0.5‐s intervals over 10 s.

### Grading of maltracking severity

**FIGURE 1:**
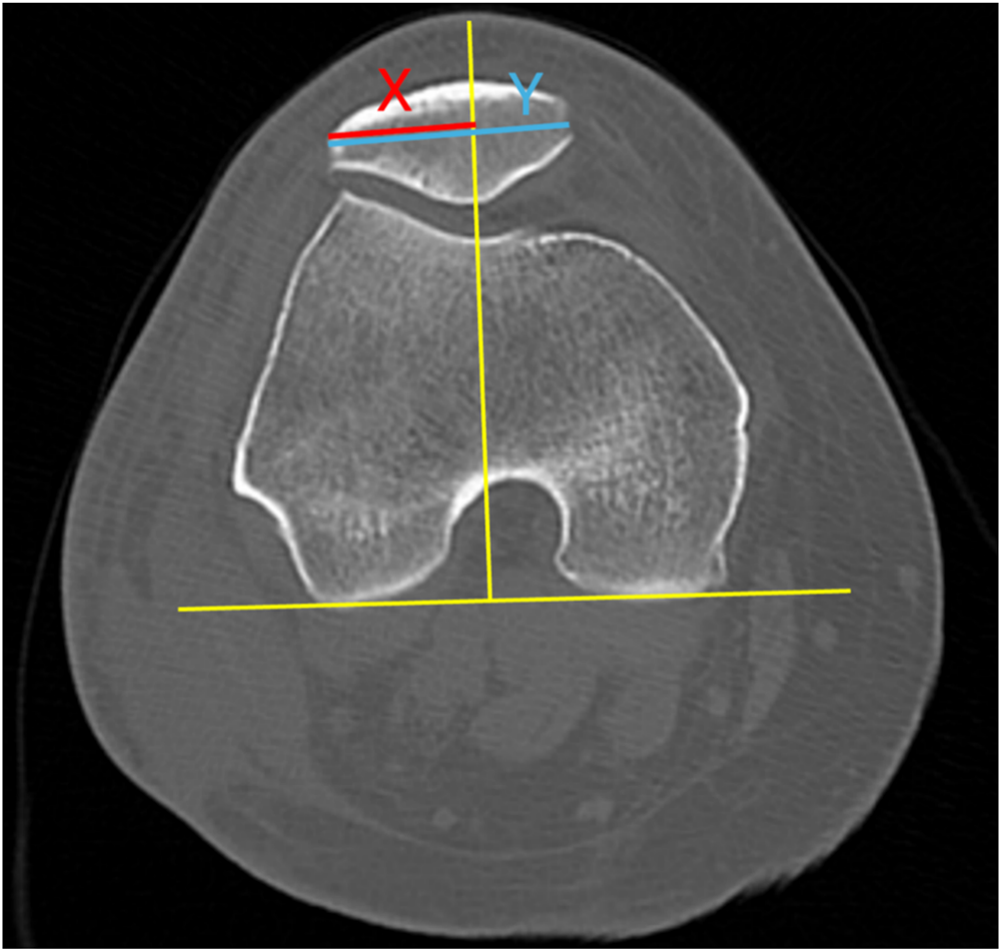
Measurement of the patellar bisect offset (BO) on an axial computed tomography image, adapted from Brossmann et al. [4]. A tangent to the posterior femoral condyles is drawn at the cut showing the perfect Roman arch. A line perpendicular to this tangent, intersecting the trochlea at its deepest point is drawn. Distances *X* and *Y* are then measured along the horizontal axis of the patella. BO (%) = *X/Y* × 100.

**FIGURE 2:**
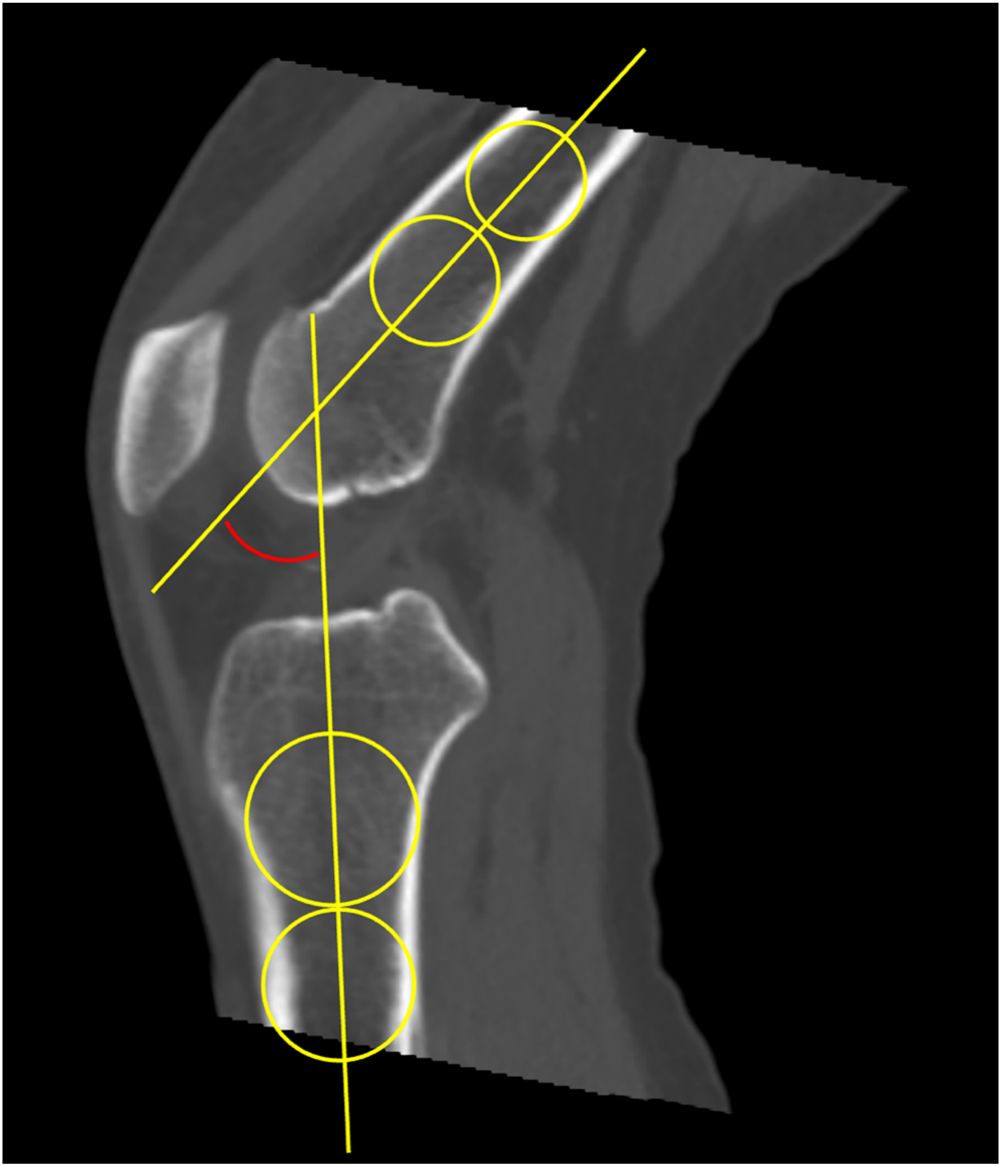
The flexion angle at each captured image was defined on sagittal dynamic kinematic computed tomography images as the angle formed by intersecting lines drawn along the centre of the femoral shaft and the tibial shaft. Hyperextension was recorded as negative value.

To objectively measure lateral patellar translation during knee range of motion, we assessed BO at 10° intervals from 0° to 50° of flexion using techniques previously described (Figure 1) [16, 18, 19]. The flexion angle at each captured image was defined on sagittal DKCT images as the angle formed by intersecting lines drawn along the centre of the femoral shaft and the tibial shaft (Figure 2). The J‐sign was measured and graded in full extension as previously described [19]. The grading system was as follows: Grade 0, <1 quadrant of lateral translation (BO, 50%–74%); Grade 1, 1 to <2 quadrants (BO, 75%–99%); Grade 2, 2 to <3 quadrants (BO, 100%–124%); and Grade 3, ≥3 quadrants (BO, ≥125%). Measurements were performed by an orthopaedic resident (M.J.B.) trained in the described techniques.

### Parameters of patellofemoral tracking

The tibial tubercle–trochlear groove distance (TTTG), lateral trochlear inclination (LTI) and Caton–Deschamps Index (CDI) were assessed according to previously described techniques [3].

### Video capture

Informed consent for video capture was obtained from all participants, as outlined in our institutional review board protocol. Videos were recorded using a mobile smartphone (iPhone 6 or 7; Apple, Inc), capturing each participant performing bilateral knee extension to document patellar tracking. Patients were instructed to replicate the motion and speed of the DKCT. Thus, patients were instructed to slowly extend and flex their knees over two cycles at an even pace over the course of 10 s. Videos were obtained from the perspective of the examiner, centred between the patient's knees at the level of their chest, taken one and a half leg lengths away from the patient. Videos focused solely on the lower limbs from the waist to the feet, excluding any identifiable information.

### Video selection

Ten videos of maltracking knees (five knees, grade 2; five knees, Grade 3) and seven videos of knees without maltracking (two knees, Grade 0; five knees, Grade 1) were selected, based on the DKCT grading.

#### Survey and participant instructions

**FIGURE 3:**
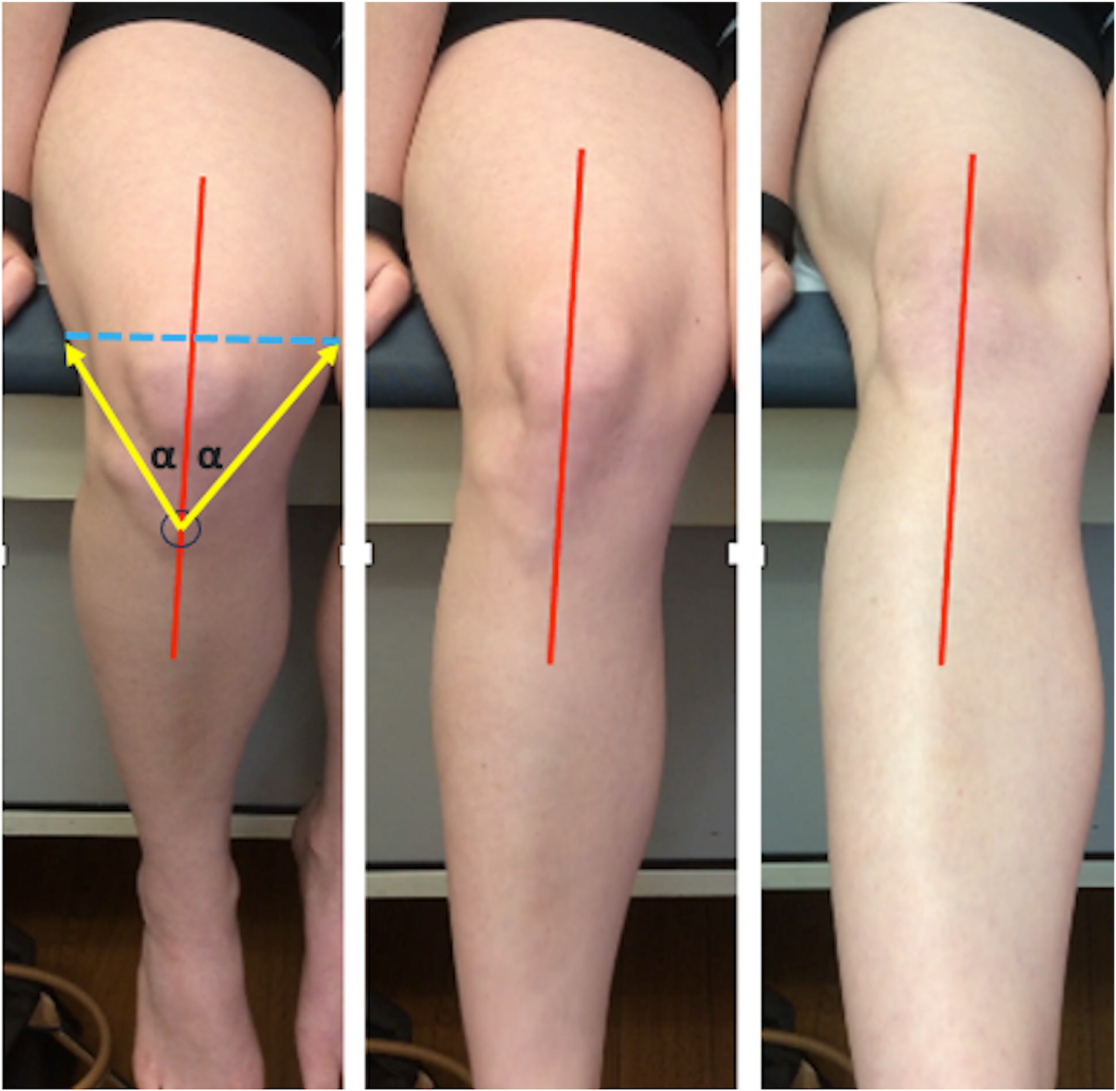
Single knee video screenshots from the examiners' perspective. A digital reference line along the centre of the femur was inserted onto the video. To create this line, the tibial tuberosity was identified with the knee at its starting point in flexion. From the tuberosity, a line bisecting the two lines from the tuberosity to the medial and lateral borders of the thigh at the level of the superior pole of the patella was inserted.

All survey participants were members of the International Patellofemoral Study Group, an international group of specialists with expertise in the patellofemoral joint. Participants reviewed all single knee videos consecutively on a large screen and were informed that some videos may repeat to evaluate intra‐rater reliability. Participants were blinded to any prior assessment in these cases. For each video, a digital reference line along the centre of the femur was inserted onto the video. To create this line, the tibial tuberosity was identified with the knee at its starting point in flexion. From the tuberosity, a line bisecting the two lines from the tuberosity to the medial and lateral borders of the thigh at the level of the superior pole of the patella was inserted (Figure 3). Lines were inserted by a fellowship trained sports medicine surgeon with experience in patellofemoral surgery and motion analysis. All videos were made to look like right knees.

All participants were asked to answer the following two questions.

1. Is the knee maltracking (>2 quadrants lateral translation in extension)?
  - No
  - Yes
2. Please grade the J‐sign:
  - Grade 0 (max translation <1 quadrant) = normal
  - Grade 1 (max translation >1 and <2 quadrants) = J1
  - Grade 2 (max translation >2 and <3 quadrants) = J2
  - Grade 3 (max translation >3 quadrants) = J3.

### Statistical analysis

#### Sample size estimate

Sample size estimates for inter‐ and intra‐observer reliability (intra‐class correlation) were calculated using G*Power 3.1.9.6 (Heinrich Heine Universität, Düsseldorf, Germany). We considered a power of 80%, a within‐rater correlation of 0.6, and a *κ* of 0.6, based on previous data [2]. Given that 17 single‐knee videos were available, the required number of raters was a minimum of 14.

#### Reliability

Inter‐rater reliability was analysed using Fleiss' kappa statistics for the qualitative (presence or absence of patellar maltracking) assessment and weighted kappa for the quantitative assessment (grading of J‐sign from 0 to 3). Intra‐rater reliability was analysed using Cohen's kappa for qualitative assessments and linear weighted kappa for quantitative assessments. Reliability was assessed as poor (*κ* < 0.20), fair (*κ* = 0.21–0.40), moderate (*κ* = 0.41–0.60), substantial (*κ* = 0.61–0.80) or near perfect (*κ* = 0.81–0.99).

#### Diagnostic performance

Accuracy was defined as the ability to correctly assess patellar tracking, including identifying normal or abnormal tracking and accurately grading the J‐sign in comparison to the assigned objective reference standard (DKCT). The accuracy of the qualitative (binary) ratings was calculated as the mean percentage of correct assessments. The accuracy of the quantitative (graded) ratings was calculated both as a percentage of correct assessments (‘raw accuracy’), and by using weighted kappa statistics to account for the ordinal nature of the scale (e.g., ‘Grade 1’ is less accurate than ‘Grade 2’ if the objective grading is ‘Grade 3’). The aggregate sensitivity, specificity, positive predictive value and negative predictive value (NPV) of all raters was calculated based on binary assessments.

#### Multivariate analysis

To determine if the accuracy of correctly diagnosing the presence of a J‐sign (binary) was affected by parameters of patellar maltracking, a mixed‐effects logistic regression model was employed including the covariates TTTG, LTI and CDI.

All statistical analyses were performed in R 4.4.1 (R Foundation for Statistical Computing).

## RESULTS

A total of 17 single‐knee videos from 14 patients with unilateral or bilateral patellofemoral instability at an age of 23.6 ± 4.3 years (mean ± SD) were assessed by 20 expert raters in the present study. Seven knees of male patients and ten knees of female patients were assessed.

### Reliability

There was moderate inter‐rater reliability regarding qualitative (binary) assessments of the presence of a J‐sign (*κ* = 0.52, 95% CI [0.48–0.55]). The inter‐rater reliability regarding quantitative (grading) assessments was substantial (*κ* = 0.62, 95% CI [0.38–0.74]).

There was a substantial intra‐rater reliability regarding qualitative (binary) assessments of the presence of a J‐sign (*κ* = 0.71 [0.61–0.81]). The intra‐rater reliability regarding quantitative (grading) assessments was substantial too (*κ *= 0.69 [0.64–0.75]) (Table 1).

**TABLE 1:** Results of the inter‐ and intra‐rater reliability analysis using Cohen's kappa for binary ratings and linear weighted kappa for ordinal ratings.

		Inter‐rater	Intra‐rater
Qualitative	Binary (no/yes)	*κ* = 0.52 [0.48–0.55]	*κ* = 0.71 [0.61–0.81]
Quantitative	Ordinal (Grade 0–3)	*κ* = 0.62 [0.38–0.74]	*κ* = 0.69 [0.64–0.75]

*Note*: Cohen's kappa (*κ*). Values in brackets represent the 95% CI.

Abbreviation: CI, confidence interval.

### Diagnostic performance

**FIGURE 4:**
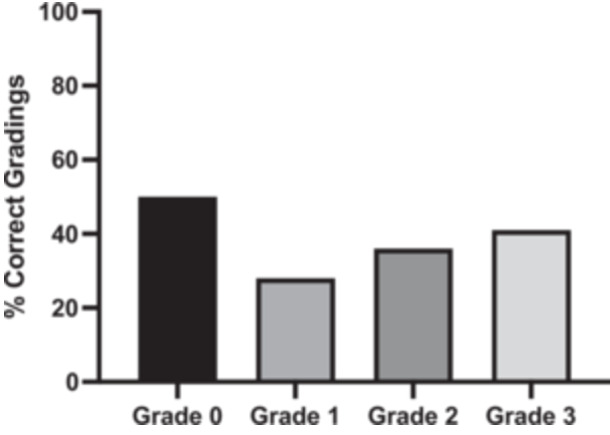
Percentage of correct assessments of the J‐sign according to grade (0–3). Only exact matches were considered correct for this analysis.

The overall mean accuracy of raters to correctly assess the presence of a J‐sign (binary) on single‐knee videos was 70.6% (Table 2). The overall mean accuracy of raters to correctly grade a J‐sign (ordinal) was 36.8%. The mean accuracy for Grade 0, 1, 2 and 3 was 50%, 28%, 36% and 41%, respectively (Figure 4).

**TABLE 2:** Aggregate diagnostic performance of binary J‐sign assessments by all raters.

Accuracy	70.6%
Sensitivity	60.5%
Specificity	85.0%
Positive predictive value	85.2%
Negative predictive value	60.1%

### Multivariate analysis

**FIGURE 5:**
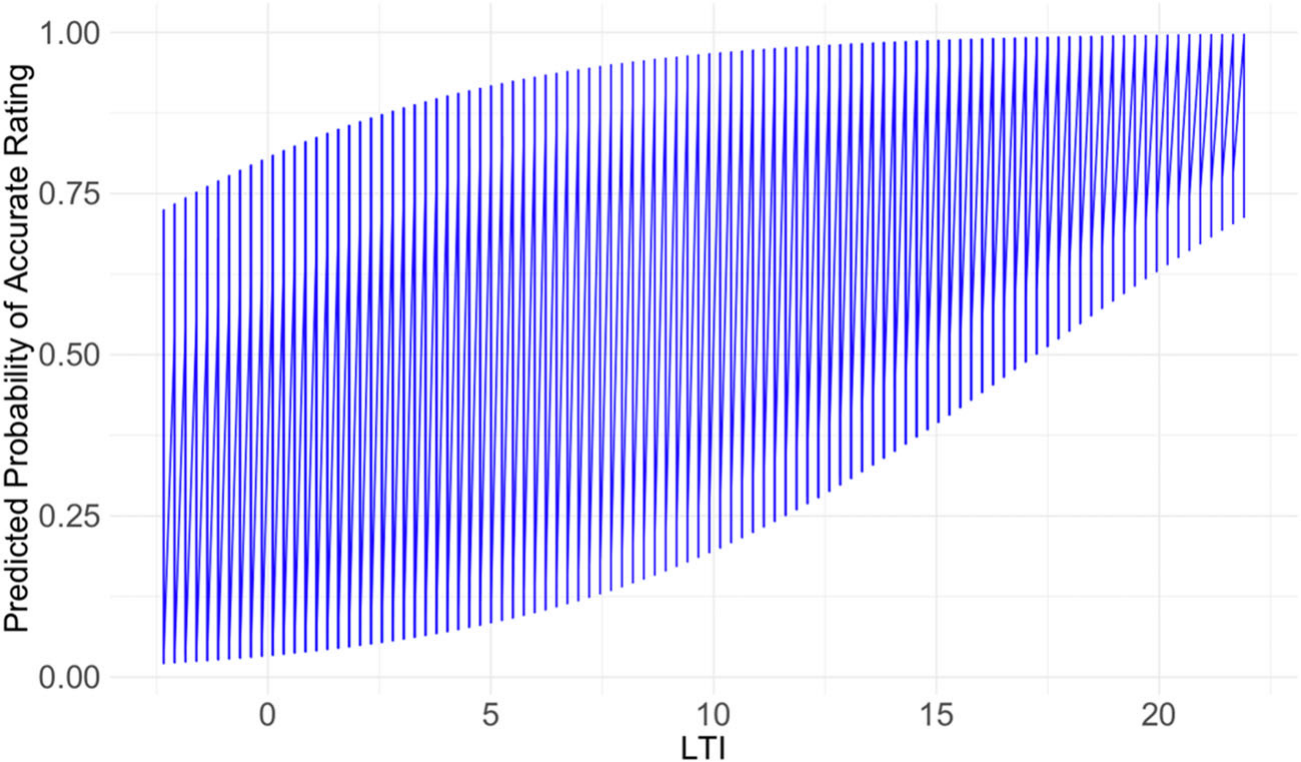
Funnel plot showing the predicted probability of correctly assessing patellar maltracking (binary) depending on the lateral trochlear inclination (LTI). A higher LTI was significantly associated with a higher probability of correctly diagnosing patellar maltracking (*p* = 0.048).

The logistic regression model demonstrated that LTI significantly predicted the accuracy of raters to correctly diagnose the presence of a J‐sign (*β* = 0.19, *p* = 0.048; Table 3). Every degree of higher LTI––indicating lesser trochlear dysplasia––was associated with an estimated 20.9% increase in accuracy (Figure 5). In knees with normal LTI (≥14, *N* = 9), the mean accuracy of binary ratings in this subgroup was 80.0% and was significantly higher compared to the overall accuracy (70.6%, *p* = 0.027). None of the other covariates (TTTG and CDI) were significantly associated with changes in accuracy.

**TABLE 3:** Multivariate regression analysis for parameters of patellofemoral tracking regarding their association with rater accuracy.

	Estimate (*β*)	SE	*p* value
Intercept	–3.37	4.40	0.444
TTTG	–0.02	0.13	0.890
LTI	0.19	0.1	0.048*
CDI (0.1 increments)	0.24	0.28	0.401

Abbreviations: *β*, estimated logistic regression coefficients; CDI, Caton–Deschamps Index; LTI, lateral trochlear inclination; TTTG, tibial tubercle–trochlear groove distance.

* *p* < 0.005.

## DISCUSSION

The present study investigated the diagnostic performance and reliability of visual assessments of patellar maltracking (J‐sign) on single knee videos with digital reference lines by patellofemoral experts in patients evaluated for patellofemoral instability. The most important finding was that the accuracy of the J‐sign was overall low and was negatively associated with decreasing LTI, indicating less accurate assessments in cases with trochlear dysplasia. Given the prognostic value of the J‐sign regarding functional outcomes and risk of recurrent dislocation, this finding is of great clinical significance [10, 11, 23, 27, 28].

### Reliability

The present data show that the inter‐rater reliability was moderate for qualitative (*κ* = 0.52) and substantial for quantitative assessments (*κ* = 0.62). In contrast to a prior analysis by Best et al., a digital reference line was employed in the present study to potentially improve the reliability of visual assessments [2]. While our study design does not allow for a definite conclusion regarding the potential benefit of this digital reference line, a between study comparison shows that the present inter‐rater reliability was higher compared to the data by Best et al. who reported a kappa of 0.45 (95% CI, 0.16–0.73) for the qualitative, and a kappa of 0.42 (95% CI, 0.14–0.48) for the quantitative assessments [2]. Also, our results showed higher inter‐rater reliability compared to a study by Hiemstra et al. [8].

In contrast, Walla et al. reported a higher inter‐rater kappa of 0.76 for qualitative (binary) assessments [21]. However, in their study, patients were classified as having either a ‘large’ or ‘small or absent’ J‐sign which may potentially have led to a more reliable cut‐off than ‘present’ versus ‘absent’. This notion is supported by the fact that grade 1 assessments were the least accurate (28%) in the current study.

The intra‐rater reliability was considered substantial, both regarding the qualitative (*κ* = 0.71) and the quantitative J‐sign assessments (*κ* = 0.69). In both cases the intra‐rater reliability was higher compared to the previously reported data by Best et al. with a kappa of 0.59 for the qualitative, and 0.48 for the quantitative assessments, pointing towards a beneficial effect of the digital reference line [2]. An even higher intra‐rater reliability was reported by Walla et al. with a kappa of 0.75 [21], which again, may underscore a better reliability when small and absent J‐signs are pooled against high‐grade ones.

### Diagnostic performance

The accuracy of 70.6% to correctly diagnose the presence or absence of a J‐sign is lower than expected, considering the high expertise among the raters and indicates a limited validity of visually assessing the J‐sign on physical examination as a parameter of patellar maltracking. Furthermore, only 36.8% of assessments regarding the grading of a potential J‐sign were accurate, which should raise concern as to whether grading the J‐sign on physical examination is reasonable. Previous data by Best et al. showed similar findings regarding the accuracy of visual J‐sign assessments [2]. With 68% of the raters correctly identifying patellar maltracking and 55% of the raters correctly grading it, the grading performance was slightly higher compared to the present study, despite the lack of a digital reference line. One possible explanation is that raters may prioritise evaluating the dynamic movement of the patella rather than its absolute lateral position. If this is the case, the presence of a static reference line may not significantly enhance the accuracy of their assessments. However, this hypothesis was not directly examined in the current study and remains beyond its scope. Future studies are needed with direct comparisons to determine the utility of a digital reference line for enhancing diagnostic accuracy, particularly by identifying specific groups in which the test is or is not reliable.

Elias et al. have reported a negative correlation between LTI and BO at low flexion angles (<20°), indicating a higher degree of patellar maltracking in patients with more pronounced trochlear dyplasia [7]. In contrast, the tibial tuberosity posterior cruciate ligament (TT‐PCL) distance was associated with maltracking both at low and higher flexion angles. In the present study, we investigated if those relationships translated into diagnostic accuracy of visual assessments of patellar maltracking. The regression model showed that only the LTI was positively associated with the raters’ accuracy, indicating that a steeper lateral inclination of the trochlea was associated with a higher probability of a correct visual assessment. This finding was confirmed in a subgroup analysis showing that the J‐sign was more accurate in patients without dysplasia (LTI ≥ 14). Hence, the validity of visually identifying a potential J‐sign may not be given in patients with a flat LTI (i.e., greater trochlear dysplasia). Several studies have demonstrated an association between a lower LTI and greater patella lateralisation measured by different imaging modalities [3, 20, 21]. While patellar maltracking might be expected to be more accurate under these circumstances, our findings suggest the opposite. A potential explanation is that the lateral trochlear facet then provides only minimal restraint, which may lead to a greater albeit more gradual lateralisation during active knee extension that may be harder to notice. In contrast, a more sudden lateralisation (i.e., ‘jumping j‐sign’) may be expected when the patella disengages from a steeper lateral trochlear facet upon full extension. These findings are corroborated by the low NPV of the visual assessments, indicating that patellar maltracking cannot be excluded based on the absence of a J‐sign on clinical examination. Therefore, surgical planning regarding realignment procedures should primarily be based on radiographic parameters rather than clinical assessment of patellar maltracking. However, the current study design does not allow for definite conclusions about these associations. Thus, future studies should investigate the dynamic component of patellar lateralisation depending on the degree of trochlear dysplasia, and its associations with visual diagnostic accuracy regarding patellar maltracking (i.e., the J‐sign).

The choice of DKCT as a comparative method for grading the J‐sign warrants consideration. Although this technique has not yet been established as the gold standard in large‐scale clinical studies, it provides a more precise and reproducible quantification of patellar tracking than visual inspection alone. Given that the J‐sign is inherently a subjective clinical finding, reliance solely on video analysis may underestimate the complexity of patellar maltracking. DKCT, in contrast, allows objective measurement of patellar lateralisation throughout knee motion.

## LIMITATIONS

This study is not without limitations. Given that patellar tracking was assessed on videos, actual assessments on physical examination including haptic feedback during manual palpation and watching the knee under active ROM from different angles may be more reliable and accurate than our findings suggest. Raters had a high level of expertise which could further undermine the generalisability of the present data and potentially represent a best‐case scenario. The experience levels of the raters were not factored in this study. Intra‐rater reliability was assessed within the first assessment run without an extended waiting period.

## CONCLUSION

Visual assessments of patellar maltracking with the use of a digital reference line demonstrated substantial reliability among a patellofemoral expert group. However, the accuracy determined by comparing the assessments with objective patella lateralisation on DKCT scans was overall low. A lower LTI was significantly associated with a decreased accuracy, indicating that visual assessments of patellar maltracking may be especially inaccurate in patients with high‐grade trochlear dysplasia.

## AUTHOR CONTRIBUTIONS

Alexander Bumberger performed the statistical analysis and drafted the manuscript. Andrew J. Cosgarea drafted the manuscript and critically reviewed the manuscript. Petri J. Sillanpaa drafted the manuscript and critically reviewed the manuscript. Matthew J. Best performed the CT‐measurements and critically reviewed the manuscript. Miho J. Tanaka drafted the manuscript and critically reviewed the manuscript.

## CONFLICT OF INTEREST STATEMENT

Alexander Bumberger has nothing to declare. Andrew J. Cosgarea has nothing to declare. Petri J. Sillanpaa: Consultant for Inion. Matthew J. Best: Other support from Arthrex. Miho J. Tanaka: Consultant for Arthrex, Vericel, Johnson & Johnson Medtech. AOSSM Board of Directors, AO North America Sports Medicine Steering Board, AJSM Associate Editor, Arthroscopy Journal Editorial Board, JBJS CME Associate Editor, Journal of Women's Sports Medicine Editor in Chief.

## ETHICS STATEMENT

The current study was approved by the Institutional Review Board at Massachusetts General Hospital, 2019P003614. Written informed consent was obtained prior to patient enrolment.

## Data Availability

The data that support the findings of this study are available from the corresponding author upon reasonable request.

## References

[1] Beckert MW , Albright JC , Zavala J , Chang J , Albright JP . Clinical accuracy of J‐sign measurement compared to magnetic resonance imaging. Iowa Orthop J. 2016;36:94–97. 27528843 PMC4910780

[2] Best MJ , Tanaka MJ , Demehri S , Cosgarea AJ . Accuracy and reliability of the visual assessment of patellar tracking. Am J Sports Med. 2020;48(2):370–375. 31913663 10.1177/0363546519895246

[3] Biyani R , Elias JJ , Saranathan A , Feng H , Guseila LM , Morscher MA , et al. Anatomical factors influencing patellar tracking in the unstable patellofemoral joint. Knee Surg Sports Traumatol Arthrosc. 2014;22(10):2334–2341. 25063490 10.1007/s00167-014-3195-y

[4] Brossmann J , Muhle C , Schröder C , Melchert UH , Büll CC , Spielmann RP , et al. Patellar tracking patterns during active and passive knee extension: evaluation with motion‐triggered cine MR imaging. Radiology. 1993;187(1):205–212. 8451415 10.1148/radiology.187.1.8451415

[5] Cao Y , Zhang Z , Shen J , Song G , Ni Q , Li Y , et al. Derotational distal femoral osteotomy yields satisfactory clinical outcomes in pathological femoral rotation with failed medial patellofemoral ligament reconstruction. Knee Surg Sports Traumatol Arthrosc. 2022;30(5):1809–1817. 34596695 10.1007/s00167-021-06739-w

[6] Dandu N , Hevesi M , Phillips AR , Haneberg EC , Elias TJ , Wang Z , et al. Anatomic drivers of J‐sign presence and severity: if there is a jump, look for a bump. Am J Sports Med. 2025;53:1119–1126. 40071349 10.1177/03635465251322788

[7] Elias JJ , Soehnlen NT , Guseila LM , Cosgarea AJ . Dynamic tracking influenced by anatomy in patellar instability. Knee. 2016;23(3):450–455. 26922799 10.1016/j.knee.2016.01.021

[8] Hiemstra LA , Sheehan B , Sasyniuk TM , Kerslake S . Inter‐rater reliability of the classification of the j‐sign is inadequate among experts. Clin J Sport Med. 2022;32(5):480–485. 36083327 10.1097/JSM.0000000000000997

[9] Milinkovic DD , Jovandic I , Zimmermann F , Balcarek P . The J‐sign and the body mass index determine the disease‐specific quality of life in patients with lateral patellar instability. Knee Surg Sports Traumatol Arthrosc. 2022;30(5):1672–1678. 34424355 10.1007/s00167-021-06705-6

[10] Milinkovic DD , Jovandic I , Zimmermann F , Balcarek P . The J‐sign and the body mass index determine the disease‐specific quality of life in patients with lateral patellar instability. Knee Surg Sports Traumatol Arthrosc. 2022;30(5):1672–1678. 34424355 10.1007/s00167-021-06705-6

[11] Pappa N , Flanigan DC , Long J , Dorweiler M , Fowler B , Duerr R , et al. Influence of patellofemoral anatomy on outcomes of isolated medial patellofemoral ligament reconstruction for recurrent patellar instability. Orthop J Sports Med. 2022;10:23259671221104414. 35783469 10.1177/23259671221104414 PMC9247377

[12] Post WR . Current concepts clinical evaluation of patients with patellofemoral disorders. Arthrosc ‐ J Arthrosc Relat Surg. 1999;15(8):841–851. 10.1053/ar.1999.v15.015084 10564862

[13] Rezvanifar SC , Flesher BL , Jones KC , Elias JJ . Lateral patellar maltracking due to trochlear dysplasia: a computational study. Knee. 2019;26(6):1234–1242. 31786000 10.1016/j.knee.2019.11.006 PMC6926151

[14] Rousseau‐Saine A , Nault ML , Hiemstra LA . What is the J‐sign and why is it important? Curr Opin Pediatr. 2023;35(1):97–101. 36592028 10.1097/MOP.0000000000001193

[15] Sappey‐Marinier E , Sonnery‐Cottet B , O'Loughlin P , Ouanezar H , Reina Fernandes L , Kouevidjin B , et al. Clinical outcomes and predictive factors for failure with isolated MPFL reconstruction for recurrent patellar instability: a series of 211 reconstructions with a minimum follow‐up of 3 years. Am J Sports Med. 2019;47(6):1323–1330. 31042437 10.1177/0363546519838405

[16] Stanford W , Phelan J , Kathol MH , Rooholamini SA , El‐Khoury GY , Palutsis GR , et al. Patellofemoral joint motion: evaluation by ultrafast computed tomography. Skeletal Radiol. 1988;17(7):487–492. 3201275 10.1007/BF00364042

[17] Tan SHS , Kwan YT , Lee JZJ , Yeo LKP , Lim AKS , Hui JH . Patellar tilt, congruence angle, and tibial tubercle‐trochlear groove distance are correlated with positive J‐sign in adolescents. Phys Sportsmed. 2024;52:492–496. 38314751 10.1080/00913847.2024.2315012

[18] Tanaka MJ , Elias JJ , Williams AA , Carrino JA , Cosgarea AJ . Correlation between changes in tibial tuberosity‐trochlear groove distance and patellar position during active knee extension on dynamic kinematic computed tomographic imaging. Arthrosc ‐ J Arthrosc Relat Surg. 2015;31(9):1748–1755. 10.1016/j.arthro.2015.03.015 25940399

[19] Tanaka MJ , Elias JJ , Williams AA , Demehri S , Cosgarea AJ . Characterization of patellar maltracking using dynamic kinematic CT imaging in patients with patellar instability. Knee Surg Sports Traumatol Arthrosc. 2016;24(11):3634–3641. 27358051 10.1007/s00167-016-4216-9

[20] Teng H‐L , Chen YJ , Powers CM . Predictors of patellar alignment during weight bearing: an examination of patellar height and trochlear geometry. Knee. 2014;21(1):142–146. 24071368 10.1016/j.knee.2013.08.011

[21] Walla N , Moore T , Harangody S , Fitzpatrick S , Flanigan DC , Duerr RA , et al. Qualitative visual assessment of the J‐sign demonstrates high inter‐rater reliability. J ISAKOS. 2023;8(6):420–424. 37499874 10.1016/j.jisako.2023.07.006

[22] Wang D , Liu Y , Sun J , Fu Q , Lv C , Yue T , et al. Patients with jumping sign exhibit rotational and bony structural abnormalities consistent with high‐grade J‐sign in recurrent patellar dislocation. Knee Surg Sports Traumatol Arthrosc. 2025;33:2379–2389. 39803705 10.1002/ksa.12584

[23] Wang D , Zhang Z , Cao Y , Song G , Zheng T , Di M , et al. Recurrent patellar dislocation patients with high‐grade J‐sign have multiple structural bone abnormalities in the lower limbs. Knee Surg Sports Traumatol Arthrosc. 2024;32(7):1650–1659. 38651601 10.1002/ksa.12186

[24] Wang D , Zheng T , Cao Y , Zhang Z , Di M , Fu Q , et al. Derotational distal femoral osteotomy improves subjective function and patellar tracking after medial patellofemoral ligament reconstruction in recurrent patellar dislocation patients with increased femoral anteversion: a systematic review and meta‐analysis. Knee Surg Sports Traumatol Arthrosc. 2024;32(1):151–166. 38226710 10.1002/ksa.12021

[25] Williams AA , Elias JJ , Tanaka MJ , Thawait GK , Demehri S , Carrino JA , et al. The relationship between tibial tuberosity‐trochlear groove distance and abnormal patellar tracking in patients with unilateral patellar instability. Arthrosc ‐ J Arthrosc Relat Surg. 2016;32(1):55–61. 10.1016/j.arthro.2015.06.037 26440373

[26] Xue Z , Song G , Liu X , Zhang H , Wu G , Qian Y , et al. Excessive lateral patellar translation on axial computed tomography indicates positive patellar J sign. Knee Surg Sports Traumatol Arthrosc. 2018;26(12):3620–3625. 29560511 10.1007/s00167-018-4897-3

[27] Zhao Z , Wang Y , Li J , Wang H , Bai X , Wang Q , et al. Clinical outcomes and prognostic factors in patients with recurrent patellar lateral dislocation treated with isolated medial patellofemoral ligament reconstruction: a retrospective single‐center analysis. Orthop J Sports Med. 2021;9:2325967121995803. 33954219 10.1177/2325967121995803 PMC8044575

[28] Zimmermann F , Milinkovic DD , Zimmerer A , Balcarek P . When should bony correction be considered in addition to medial patellofemoral ligament reconstruction? results of a clinically derived 2‐group classification of lateral patellar instability based on 122 patients at 2‐ to 5‐year follow‐up. Orthop J Sports Med. 2023;11:23259671221147572. 36743734 10.1177/23259671221147572 PMC9893382

